# Case Report: Chemotherapy and Radiotherapy Combined With DC-CIK for Pulmonary and Mediastinal Metastases From Nasopharyngeal Carcinoma

**DOI:** 10.3389/fonc.2022.778643

**Published:** 2022-02-17

**Authors:** Yi-Xiu Gan, Gui-Hua Li, Xue Ou, Chun-Hui Wang, Qing-Hua Du

**Affiliations:** Department of Radiation Oncology, Second Affiliated Hospital of Guangxi Medical University, Nanning, China

**Keywords:** cytokine-induced killer cell, radiotherapy, chemotherapy, nasopharyngeal carcinoma, pulmonary and mediastinal metastasis

## Abstract

**Introduction:**

The optimal treatment for pulmonary and mediastinal metastasis of nasopharyngeal carcinoma (NPC) is still controversial, and the therapeutic effect is poor recently. In one case, we demonstrated a long-term survival after postoperative chemoradiotherapy combined with dendritic cell and cytokine-induced killer (DC-CIK) immunotherapy for pulmonary and mediastinal metastases from NPC.

**Baseline Characteristics:**

A 53-year-old woman was admitted to our hospital in June 2008. Pathological biopsy revealed a poorly differentiated squamous cell carcinoma located in the nasopharynx with the invasion of internal pterygoid muscles, the sphenoid bone, and unilateral neck lymph node metastasis. No distant metastases were observed. The stage of NPC was T3N1M0 III (AJCC8). The patient received concurrent chemoradiotherapy for primary lesion and neck lymph nodes and achieved complete remission (CR) of the disease after 3 months. Follow-up at 3-month intervals was carried out. Pulmonary and mediastinal lymph node metastases were found in July 2009. The patient then underwent right upper lobectomy and mediastinal lymph node dissection and five cycles of gemcitabine and cisplatin (GP) regimen chemotherapy, following radiotherapy and DC-CIK immunotherapy.

**Results:**

After a follow-up time of 13 years, no tumor recurrence or metastasis and severe adverse reactions were found.

**Conclusion:**

Postoperative chemotherapy and radiotherapy in combination with DC-CIK immunotherapy may produce a synergistic therapeutic effect on patients with mediastinal lymph node metastasis from NPC.

## Introduction

With continuous technological improvements in radiotherapy, the locoregional control and survival rates of nasopharyngeal carcinoma (NPC) have improved and the 5-year local recurrence rate for newly diagnosed and nonmetastatic NPC has been reduced to 7.4% (1). The main failure mode is distant metastasis (2). Patients with recurrent or metastatic diseases have a poor prognosis, with a median survival of only about 20 months (3). The incidence of NPC with pulmonary and mediastinal metastases is extremely low (4). Currently, there is no standard treatment available. Immunotherapy is one of the systematic treatments for metastatic disease. Since cytokine-induced killer (CIK) cells were first reported by Schmidt-Wolf (5), dendritic cell and cytokine-induced killer (DC-CIK) therapy has shown good clinical application prospects due to its strong antitumor activity, and it has rapidly become a new hot spot of antitumor biological adoptive immunotherapy. We reported a case of a patient with mediastinal metastasis from NPC. The result suggested that the combination of postoperative radiotherapy and chemotherapy along with immunotherapy could bring a long-term survival for the patient with mediastinal lymph nodes from NPC.

## Case Presentation

### Baseline Characteristics of the Patient

**Figure 1:**
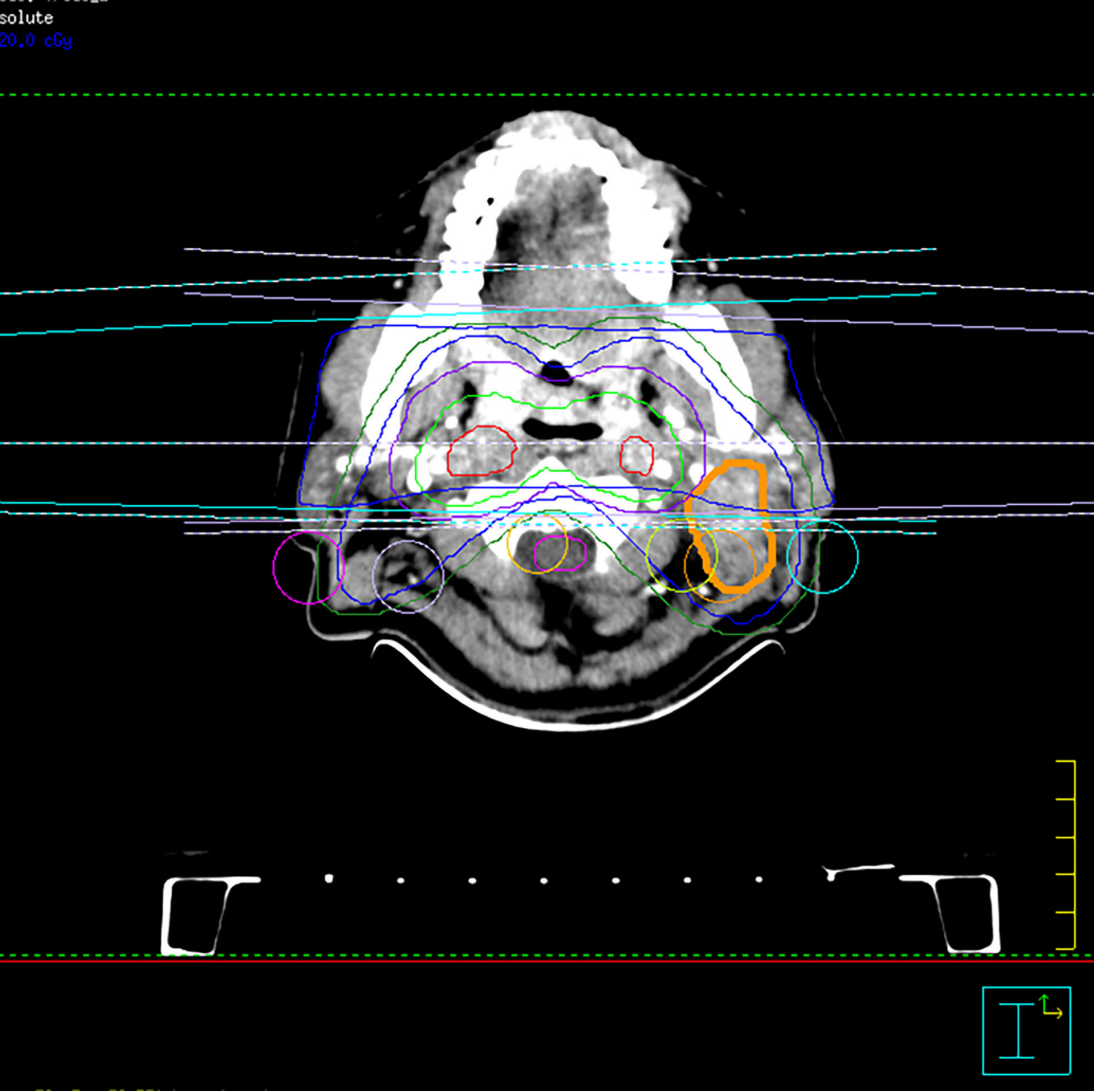
Radiation for nasopharyngeal primary lesion and positive cervical lymph nodes.

A 53-year-old woman was admitted to the Second Affiliated Hospital of Guangxi Medical University in June 2008. Nasopharyngoscope examination revealed that the tumor was located in the nasopharynx, invading the bilateral pharyngeal recess and the pharyngeal opening of the Eustachian tube, which was later pathologically confirmed to be a poorly differentiated squamous cell carcinoma. Computed tomography (CT) scan showed that the tumor had invaded the skull base and the patient had bilateral retropharyngeal lymph nodes (RPLN) and levels II–III lymph node metastasis in her left neck. The longest of the short-axis diameter of lymph nodes was about 2.5 cm. No distant metastasis was found. The disease was diagnosed as stage III (T3N1M0) in the 8th edition of the American Joint Committee on Cancer (AJCC8) Staging Manual (**Figure 1**).

### Initial Treatment

Treatment was performed with curative conventional fractionated radiotherapy at a dose delivered to the plan gross tumor volume of the nasopharynx (PGTVnx) and gross tumor volume of the lymph nodes (PGTVnd) of at least 7,000 cGy in 35 fractions, with 200 cGy once a day and 5 times per week, followed by application of the shrinking-field technique to limit irradiation of the spinal cord to a maximum dose of 4,000 cGy. The preventive dose of 5,500 cGy/28 fractions was prescribed to the lower-risk clinical target volume (CTV). The PGTVnx included the nasopharynx and the area directly invaded by the tumor including the internal pterygoid muscle and the skull base. The patient received 2 cycles of PF regimen (fluorouracil 1,000 mg/m²d1–d4+cisplatin 80 mg/m²/d1) concomitant chemotherapy and achieved complete remission (CR) of the disease after 3 months.

### Pulmonary and Mediastinal Metastasis and Treatment

**Figure 2:**
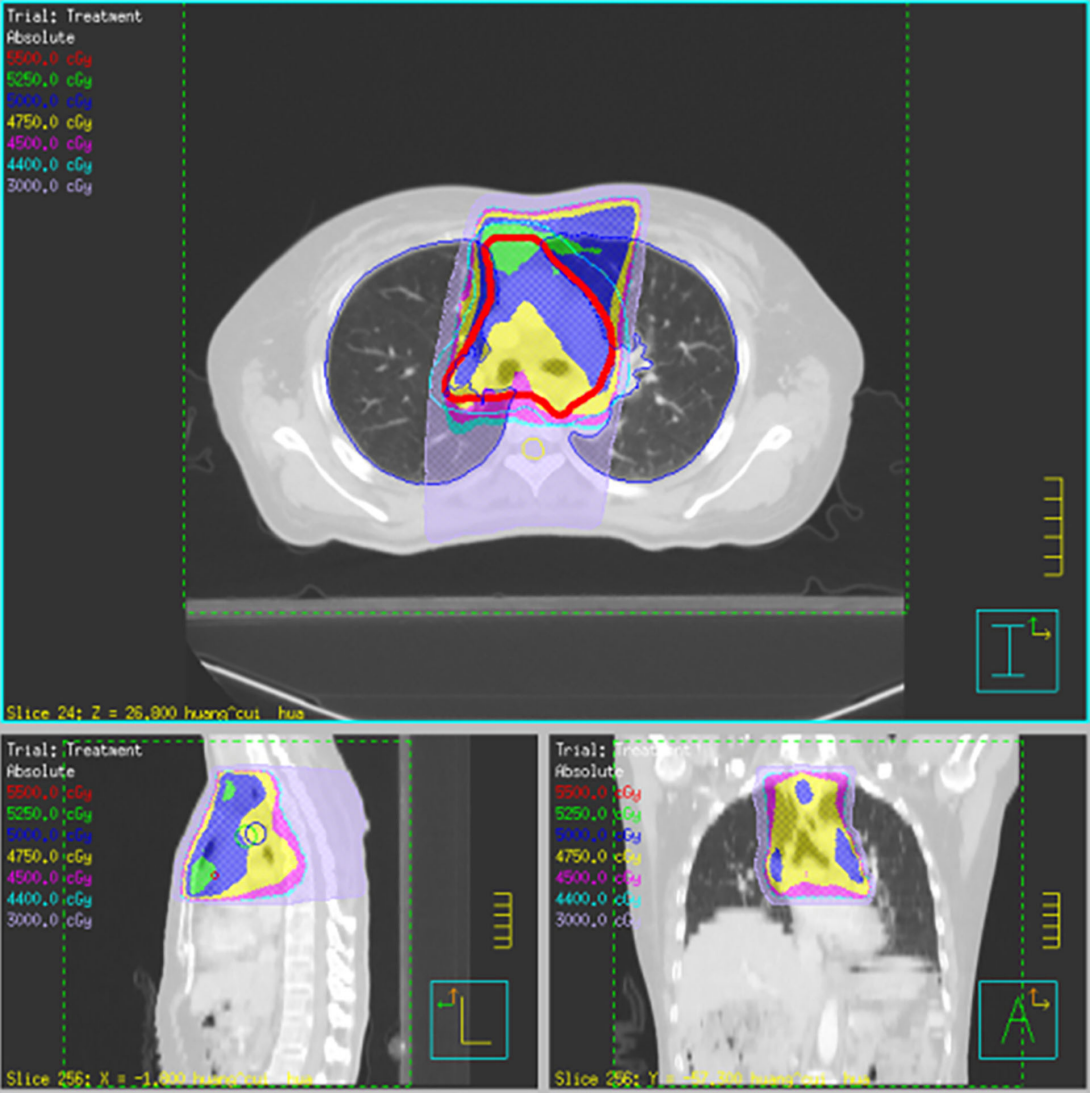
Postoperative radiotherapy for mediastinum.

CT scan of the chest revealed metastasis to the right upper lung with a tumor diameter of 1 cm and mediastinal lymph node metastasis in July 2009. The patient underwent right upper lobectomy and mediastinal lymph node dissection on July 20, 2009. Postoperative pathology and immunohistochemistry indicated that the right upper pulmonary nodule was a poorly differentiated squamous cell carcinoma and the margin of bronchial stump was negative. Three (3/17) lymph node metastases were found in two levels of the drainage area of the mediastinum, in which two (2/6) were upper paratracheal lymph nodes and one (1/3) was a lower paratracheal lymph node. Subcarinal, pulmonary ligament, and hilar lymph nodes were negative for metastasis. The patient received 5 cycles of GP regimen (gemcitabine 1,000 mg/m² on the 1st and 8th days and cisplatin 80 mg/m² on the first day) from August to November 2009. The treatment was repeated every 3 weeks. Postoperative radiotherapy was processed from December 2009 to February 2010. Conventional fractionated radiotherapy for mediastinum was performed at a dose of 5,000 cGy in 25 fractions (**Figure 2**). The major adverse reactions during chemoradiotherapy were grades 1–2 myelosuppression and digestive tract reactions. DC-CIK immunotherapy was started 3 weeks later. The patient was injected with allogeneic CIK cells (1*10^9^/250 ml, once a week for 5 weeks). The immunotherapy process includes the following steps: First of all, 100 ml of peripheral blood (anticoagulation) was extracted from the patient, and mononuclear cells were separated and collected by blood cell separator for DC and CIK culturing. After 7 days of culture, mature DC and CIK were collected and were mixed at a ratio of 1:100 for reculture for 7 days. During this period, sterility test was carried out. The survival rate of cells was guaranteed to be above 95%. DC-CIK therapy was started 3 weeks later after radiotherapy, and intravenous infusion of DC-CIK mixed suspension (1 × 10^9^ each time) was performed for a total of 5 times. The primary toxicities of immunotherapy were grade II granulocytopenia, grade I dry skin, and low-grade fever. After the completion of the therapy, oncology follow-up was carried out every 2–3 months during year 1, every 3–4 months during years 2–3, then every 6 months during years 4–5, and then annually. A 13-year follow-up was then conducted. The patient achieved a long-term survival of 13 years without recurrence or metastasis. In June 2011, tests revealed paralysis of the vocal cords. After radiotherapy, the patient developed a dry mouth. Long-term follow-up showed a small amount of pleural effusion and pleural hypertrophy, but it did not affect normal respiratory function and daily life.

## Discussion

With the deepening of research, immunotherapy has become a new and hot treatment and developed into one of the four largest treatments for cancer (6). Recently, basic research and some clinical trials showed that the combination treatment of chemotherapy, radiotherapy, and immunotherapy could promote an antitumor effect of the body’s immune system and improve the therapeutic effect for patients (7).

DC-CIK adoptive immunization is one of the important means of immunotherapy. CIK cells are a kind of heterotypic cells with different cell phenotypes obtained by the co-culturing of human peripheral blood monocytes and various cytokines. It is highly lethal to tumor cells. Its main effector cells are CD3+ and CD56+ cells. It has strong antitumor activity of T lymphocytes and non-MHC restricted tumor-killing characteristic of NK cells. It has been proved that CIK cells are mainly derived from CD4− CD8+ T lymphocytes, namely cytotoxic T lymphocytes (CTL), which mainly play a specific direct killing effect, so the killing mechanism of CIK cells is similar to that of CTL (8). CIK cells can kill tumor cells directly through the lymphocyte function-associated antigen-1/intercellular adhesion molecule-1 (LFA-1//ICAM-1) pathway. DC are the most powerful antigen-presented cells (antigen-presenting cell (APC)). Its mechanisms include the following: DC presents exogenous antigens to CD3/CD4 and CD3/CD8 T lymphocytes through non-MHC-I and non-MHC-II pathways, inducing body to produce and release antigen-specific T lymphocytes, thus playing a role in recognition and killing. It also can stimulate the immune memory function of the body and quickly plays a protective role when it is attacked by the same antigen again (9). Mixed co-culture of the two can promote the maturity of DC, secrete costimulatory molecules and cytokines, activate CIK, increase the proliferation ratio, and improve the destruction of tumor cells. It promotes the proliferation of antigen-specific T cells and plays a role in long-term immune surveillance of tumors.

In this case, DC-CIK immunotherapy was performed after radiotherapy. Previous studies showed that local radiotherapy combined with immunotherapy could produce an immune-enhancing antitumor mode mediated by radiotherapy. The main mechanisms are as follows: Firstly, radiotherapy can promote the amplification and recognition of antigens. The strength of antitumor immune effect is closely related to the effective recognition and presentation of antigens. It is well known that antigen recognition and presentation is the first step of the body’s immune response. The class I of major histocompatibility complex-1 (MHC-1) is a key molecule to recognize antigens for CD8+ T cells. The expression of MHC-1 was significantly inhibited in tumor tissues (10). Radiotherapy can promote the expression of MHC-1 molecule (11), and local high-dose radiotherapy can enhance antigen presentation of DCs (12). At the same time, radiotherapy induced the decomposition and rupture of tumor cells, and the released high-mobility group box 1 protein (HMGB-1) molecule could bind to toll-like receptor-4 (TLR-4) molecule, thereby promoting the expression of MHC-1 (12). It was also found that the HMGB-1 content in blood increased significantly in patients with esophageal cancer after radiotherapy (13). Secondly, radiotherapy promotes immune response. The promotion of radiotherapy on the immune response of the body is mainly mediated by CD8+ T cells. Animal experiments have proved that the strength of the “distal effect” is related to the number of infiltrated CD8+ T cells. After receiving radiotherapy, the number of T cells in the adjacent lymph nodes of tumor tissues is significantly higher than that of the patients without radiotherapy (14). Other studies have found that tumor-specific cells, especially CD8+ T cells, must reach a certain concentration in order to effectively inhibit the proliferation of tumor cells (15). The direct killing of tumor cells by radiotherapy induces the formation of an “*in situ* vaccine,” which activates the body’s original immune response (16). DC can present the necrotic cell products, such as DNA fragments, to CD8+ T cells as antigens. Radiotherapy leads to cell necrosis and DNA damage, which can enhance antitumor immune effect (17–19). Radiotherapy can also enhance the activation and maturation of DC by promoting the release of HMGB-1, thus enhancing the presentation of tumor antigens and promoting antitumor immune response. Simultaneously, it was found that radiation could promote the production of interleukin-1β (IL-1β) and tumor necrosis factor-α (TNF-α), and thus enhance the production of prostaglandin E2 and further promote the maturation of DC (20). Therefore, in this case, the combination of the two had a favorable effect on the treatment of this patient.

The combination of chemotherapy and immunotherapy has been shown to improve the effectiveness of cancer treatment in some cases. Some studies confirmed that some drugs, such as oxaliplatin and fluorouracil, could induce immunogenic cell death and antitumor immune response (21–26). Gemcitabine was found to not only increase the types of antigenic epitopes to trigger stronger immune response while killing tumor cells (25) but to also reduce the number of MDSCs and the secretion of immunosuppressive cytokines, at the same time, enhance the functions of killer T cells and NK cells (27). Cisplatin can also promote the expression of man-nose-6-phosphate receptor (M6PR) on the surface of tumor cells, and then increase the cell penetration of granzyme-B and the function of killer T cells (22). However, there were also other studies with opposite results. They confirmed that chemotherapy can directly kill immune cells or inhibit the immune function of natural killer (NK) cells, which has a negative effect on immunotherapy. Therefore, the mechanism of chemotherapy-immune interaction is complex, and its effect is not very clear (27–31). In this study, GP chemotherapy was performed after surgery which effectively controlled the potential distant metastatic subclinical lesions. Although the mechanism of chemotherapy combined with immunotherapy in this case could not be further traced and clarified, the comprehensive treatment mode enabled the patient to achieve long-term survival, which is worthy of further study and discussion.

The limitations of this case are mainly as follows: Firstly, although this case has achieved long-term survival, it is a single case, which is not universally representative. Secondly, there is no standard treatment mode of immunotherapy combined with radiotherapy or chemotherapy at present, and its combined effect needs further studies to clarify. Thirdly, the patient was followed up retrospectively, and the activity of immune cells cannot be accurately detected. With further research, the mechanism of radiotherapy or chemotherapy combined with immunotherapy will be further explored.

## Conclusion

In this case, the postoperative chemotherapy and radiotherapy combined with DC-CIK immunotherapy achieved a good therapeutic effect on patients with pulmonary and mediastinal lymph nodes metastases from NPC, showing that the combination of chemotherapy, radiotherapy and immunotherapy may be a potentially safe and effective systemic treatment for distant metastases of NPC. Overall, chemotherapy and radiotherapy combined with immunotherapy may enhance the antitumor effect on mediastinal metastasis of NPC.

## Data Availability Statement

The original contributions presented in the study are included in the article/supplementary material. Further inquiries can be directed to the corresponding authors.

## Ethics Statement

The studies involving human participants were reviewed and approved by the Ethics Committee of the Second Affiliated Hospital of Guangxi Medical University. The patients/participants provided their written informed consent to participate in this study. Written informed consent was obtained from the individual(s) for the publication of any potentially identifiable images or data included in this article.

## Author Contributions

G-HL collected data. C-HW followed up the patient. Y-XG and XO wrote the paper. Q-HD and Y-XG inspected the manuscript critically and took part in the revision of the manuscript. All authors have read and approved the final manuscript.

## Funding

This study was sponsored by the Scientific Foundation of the Second Affiliated Hospital of Guangxi Medical University (Grant/Award Numbers: EFYKY2020018 and 2020008).

## Conflict of Interest

The authors declare that the research was conducted in the absence of any commercial or financial relationships that could be construed as a potential conflict of interest.

## Publisher’s Note

All claims expressed in this article are solely those of the authors and do not necessarily represent those of their affiliated organizations, or those of the publisher, the editors and the reviewers. Any product that may be evaluated in this article, or claim that may be made by its manufacturer, is not guaranteed or endorsed by the publisher.
